# The selection of naturally stable candidate foot-and-mouth disease virus vaccine strains for East Africa

**DOI:** 10.1016/j.vaccine.2021.07.001

**Published:** 2021-08-16

**Authors:** Ben Jackson, Yongjie Harvey, Eva Perez-Martin, Ginette Wilsden, Nicholas Juleff, Bryan Charleston, Julian Seago

**Affiliations:** a The Pirbright Institute, Woking, Surrey GU24 0NF, United Kingdom; b Bill & Melinda Gates Foundation, 500 5th Ave N, Seattle, WA 98109, United States

**Keywords:** Foot-and-mouth disease, FMD vaccine, Foot-and-mouth disease virus, Strain stability, Virus neutralisation test

## Abstract

Foot-and-mouth disease (FMD) is a global burden on the livestock industry. The causative agent, FMD virus (FMDV), is highly infectious and exists in seven distinct serotypes. Vaccination remains the most effective control strategy in endemic regions and current FMD vaccines are made from inactivated preparations of whole virus. The inherent instability of FMDV and the emergence of new strains presents challenges to efficacious vaccine development. Currently, vaccines available in East Africa are comprised of relatively historic strains with unreported stabilities. As an initial step to produce an improved multivalent FMD vaccine we have identified naturally stable East African FMDV strains for each of the A, O, SAT1 and SAT2 serotypes and investigated their potential for protecting ruminants against strains that have recently circulated in East Africa. Interestingly, high diversity in stability between and within serotypes was observed, and in comparison to non-African A serotype viruses reported to date, the East African strains tested in this study are less stable. Candidate vaccine strains were adapted to propagation in BHK-21 cells with minimal capsid changes and used to generate vaccinate sera that effectively neutralised a panel of FMDV strains selected to improve FMD vaccines used in East Africa. This work highlights the importance of combining tools to predict and assess FMDV vaccine stability, with cell culture adaptation and serological tests in the development of FMD vaccines.

## Highlights

• Development of improved FMD vaccines for East Africa.
• Selection of candidate FMDV vaccine strains based on stability and serology.
• Stability diversity between and within FMDV serotypes.

## 1 Introduction

Foot-and-mouth disease virus (FMDV) causes one of the most highly contagious and economically devastating diseases of cattle, sheep, goats and pigs (livestock). The disease, characterised by fever and lesions inside the mouth and on the feet, does not have a high mortality rate, but morbidity rates up to 100% severely damage draught power, as well as milk and meat production. FMD is endemic in many developing regions of Asia, Africa and South America, where smallholders are particularly affected by FMD and losses in livestock productivity increases poverty.

FMDV is a member of the *Picornaviridae* family and exists as seven distinct serotypes, namely A, O, C, Asia 1 and Southern African Territories (SAT) 1, 2 and 3, with numerous subtypes within each serotype [1]. Vaccination remains the most effective tactic for controlling FMD and current FMD vaccines are made from inactivated preparations of whole virus, that must contain high levels of intact viral capsid to elicit protective immune responses. Unfortunately, the FMDV capsid readily dissociates under mild acidic conditions (pH < 7) and at elevated temperatures (>30 °C), and the inactivation process carried out during vaccine production increases this instability. This issue is further compounded by cold chain limitations and the divergence in stability observed within each serotype.

Although SAT3 is present, there are four predominant FMDV serotypes (A, O, SAT1 and SAT2) in East Africa (World Reference Laboratory for Foot-and-Mouth Disease (WRL-FMD)). Vaccination against one serotype does not provide efficacious cross protection to another serotype, and often not to disparate strains within the same serotype. However, intra-serotype protection can be implemented, particularly when informed by vaccine matching. Currently, the multivalent FMD vaccines that are available in East Africa are comprised of relatively historic strains with unreported stabilities [2], [3]. Therefore, there is an opportunity to develop improved FMD vaccines for East Africa, that have characterised thermostabilities and are better matched to recently circulating East African strains.

As an initial step to produce an improved multivalent FMD vaccine for protecting ruminants in East Africa we have successfully implicated thermofluor-based screening to identify naturally stable East African FMDV strains for each of the A, O, SAT1 and SAT2 serotypes. Candidate vaccine strains selected from these were adapted to grow in baby hamster kidney-21 (BHK-21) cells and small-scale vaccine preparations produced to generate vaccinate sera that effectively neutralised a panel of FMDV strains selected to improve FMD vaccines used in East Africa. Interestingly, we report high diversity in stability between and within serotypes and show that in comparison to non-African A serotype viruses reported to date, the East African strains tested in this study are less stable.

## 2 Materials and methods

### 2.1 Genome amplification and sequencing

Total RNA was extracted using QIAamp Viral RNA Mini Kit (Qiagen, UK) and the respective region of the viral RNA genome was reverse transcribed using SuperScript® III Reverse Transcriptase (ThermoFisher Scientidic, UK) and then amplifed by PCR using *Pfu* DNA polymerase (Thermofisher Scientific, UK) and the following pair of primers: OFiveF, 5′ cagaaccagtcaggcaacactg 3′; NK72R, 5′ gagtccaaccctgggcccttc 3′. Sequencing reactions were performed using the BIG Dye Terminator v3.1 cycle sequencing kit (Applied Biosystems, UK). Phylogeny analyses was performed using online NGphylogeny.fr software (Laboratory of Computer Science, Robotics and Microelectronics of Montpellier (LIIMM), France). MUSCLE multiple sequence alignment (EMBL-EBI) software was used to determine amino acid percent identity.

### 2.2 Viruses and cell culture

All candidate FMDV strains were purchased from the WRL-FMD as a glycerol stock with a documented passage history. ZZ-R 127 goat epithelium cells were cultured in Dulbecco's Modified Eagle Medium/Nutrient Mixture F-12 (DMEM/F12; Thermofisher Scientific, UK) and BHK-21 cells in Glasgow’s minimal essential medium (GMEM; Thermofisher Scientific, UK), with each medium supplemented with 10% adult bovine serum (penicillin (100 SI units/ml), and streptomycin (100 μg/ml).

### 2.3 Virus inactivation and purification

Following cytopathic effect (CPE) of infected cells, virus in clarified supernatants was either not inactivated or chemically inactivated by two consecutive incubations with binary ethyleneimine (BEI) at a final concentration of 0.001 M, each at 37 °C for 24  h. Live virus/inactivated antigen was then precipitated with 7.5% (w/v) PEG 6,000, resuspended in PBS, centrifuged at 2060*g* for 15 min at 4 °C and pelleted over a 30% sucrose cushion by centrifugation at 104,000*g* for 2.5 h at 12 °C. Pellets were resuspended in PBS/0.5% (v/v) IGEPAL CA-630 (Sigma Aldrich, UK), overlayed onto a 15–30% sucrose gradient and then fractionated by centrifugation at 104,000*g* for 3 h at 12 °C. Pellets were resuspended in PBS and their concentration was determined spectrophotometrically using the following formula: (OD260 × Total volume)/7.6 = mg of virus. Purified live virus and inactivated antigen were stored at 4 °C until use.

### 2.4 Thermofluor PaSTRy assay

Thermofluor PaSTRy assays (herein termed thermofluor assays) were performed in PBS buffer, or the indicated cell culture medium, using 0.1–0.4 μg of virus and SYBR green-II dye (Thermofisher Scientific, UK) at a final dilution 1:1000 and a MX3005 PCR machine (Agilent Technologies, UK) as previously described [4]. Assay temperature was ramped from 25 °C to 94 °C in 0.5 °C increments with intervals of 10 s and fluorescence was read with excitation and emission wavelengths of 490 nm and 516 nm, respectively. Data sets exported from the qPCR machine were visualized using MxPro sofware (Stratagene). Three independent thermofluor assays were performed for each analysis.

### 2.5 Vaccine preparation and vaccination of cattle with FMD vaccines

Animals were acclimatised for 7 days prior to the start of the study. Each of four groups of five male Holstein Friesian calves (100- to 150-kg) were vaccinated by intramuscular injection with 2 ml of formulated O/ETH/29/2008, A/ETH/9/2008, SAT1/KEN/80/2010 or SAT2/ETH/65/09 vaccine; each vaccination was comprised of 1 ml of respective inactivated antigen (12 µg) and 1 ml of oil adjuvant ISA201 (SEPPIC). Booster vaccines, prepared in the same way from the same inactivated antigens, were administered on day 14 of the study after sera collection. All animals were bled to collect serum on days 0, 7, 14 and 21, and virus-neutralizing-antibody titres (VNT) were determined. Animal experimentation was approved by the Pirbright Institute Ethical Review Board under the authority of a Home Office project license (70/8958) in accordance with the Home Office Guidance on the Operation of the Animals (Scientific Procedures) Act 1986 and associated guidelines. All studies complied with the Council Directive 86/609/EEC on the approximation of laws, regulations and administrative provisions of the Member States regarding the protection of animals used for experimental and other scientific purposes. Group sizes for study were consistent with those in previously published studies used to determine the induction of antibody titres consistent with protection. Animals were distributed within the groups by random permutations using an online research randomizer program (www.randomizer.org).

### 2.6 Titration of neutralizing antibodies

Serum samples collected on days 0-, 7-, 14- and 21-days post vaccination were examined for anti FMDV neutralising antibodies using the virus neutralisation test (VNT) and the corresponding homologous virus. Heterologous VNT using non-homologous viruses from the same serotype were also performed as discussed. VNTs were performed using BHK-21 cells and unless indicated clarified stocks of unpurified virus according to the protocol recommended by the World Organisation for Animal Health (Office International des Epizooties (OIE)) [5], [6]. Sera were inactivated at 56 °C for 30 min before use. Neat serum stocks were initially diluted 1:8 and then in two-fold dilutions for the tests (1:8, 1:16, 1:32, 1:64, 1:128, 1:256, 1:512, 1:1024). For each test a 100 TCID_50_ of virus was used in a total volume of 50 μl. Neutralizing antibody titres, calculated by the Spearmann-Karber method, were expressed as the last dilution of serum that neutralizes 50% of the virus [7]. r_1_ values were calculated using the formula:

$$Mean\;r_{1}\;=\;Mean\;hetero\log ous\;virus\;neutrali\sin g-antibody\;titre/\;Mean\;homo\log ous\;virus\;neutrali\sin g-antibody\;titre$$

## 3 Results

### 3.1 Selection of candidate FMD vaccine seedstock strains

**Supplementary Fig. 1:**
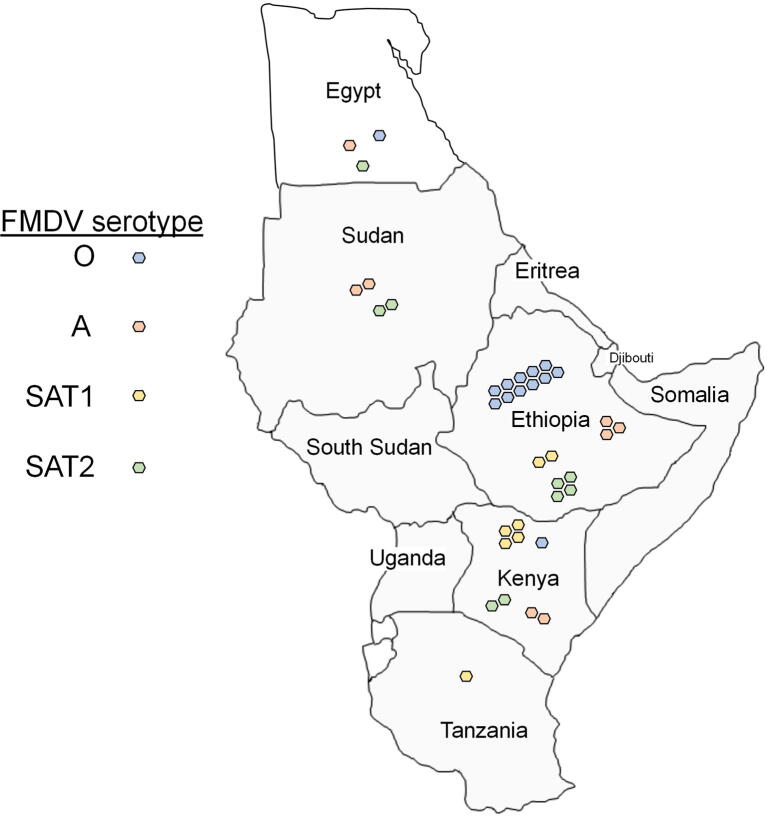
FMDV strain origin. Schematic representation of the 37 African FMDV strains (see Table 1) belonging to the O, A, SAT1 and SAT2 serotypes that were screened for thermostability. East African countries are shaded grey and Egypt (North Africa) is unshaded.

Candidate seedstock strains were selected based on their serotype (A, O, SAT1 and SAT2), subtype, the country, region and species (cattle) from which they originated, and the date they were collected (Table 1 and Supplementary Fig. 1). Predominance was given to strains derived from cattle in Ethiopia, and neighboring countries Kenya and Sudan, but included a O, A and SAT2 strain from Egypt and a SAT1 strain from Tanzania (Table 1). With regards to their lineages, viruses belonging to the O serotype comprised of topotypes East Africa 2, 3 and 4, A serotype of genotypes I, IV and VII, SAT1 serotype of topotypes I and IX, and SAT2 serotype of topotypes IV, VII, IX and XIII. With the exception of three SAT2 strains (SAT2/KEN/3/57, SAT2/KEN/52/84 and SAT2/ETH/1/90) all viruses were deposited into the WRL-FMD between 2005 and 2013. More strains were selected for FMDV O due to a larger repository collection of this serotype. In total, 37 strains were selected; consisting of 13 strains belonging to the O serotype, 8 to A, 7 to SAT1 and 9 to SAT2.

**Table 1:** FMDV vaccine strains screened for thermostability. The thermostability of 37 African FMDV strains belonging to the O, A, SAT1 and SAT2 serotypes were analysed by thermofluor assay. The respective name, lineage, country (see Supplementary Fig. 1), species of origin, and capsid dissociation temperature (*T*_r_) are shown. Control, non-African thermostable A serotype strains are listed in grey. Thermofluor assays were performed in triplicate.

### 3.2 Thermostability screening of selected viruses

**Supplementary Fig. 2:**
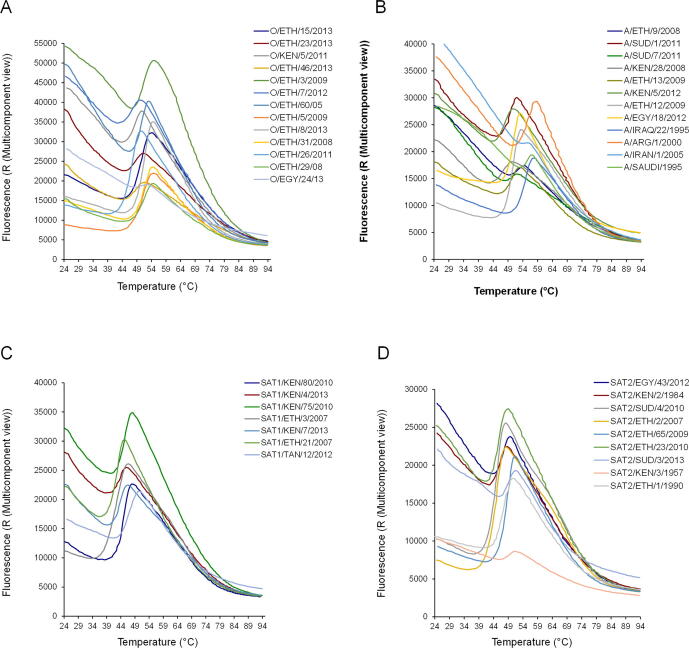
Thermofluor-based screen for candidate FMDV strains with improved thermostability. The 37 FMDV strains listed in Table 1 were screened by thermofluor assay to determine the temperature at which the viral genome is released as a result of capsid dissociation. The thermofluor plots for each of the O, A. SAT1 and SAT2 candidate vaccine strains are shown. Four non-African FMDV belonging to the A serotype (A/IRAQ/22/1995, A/IRAN/1/2005, A/ARG/1/2000 and A/SAU/1995) are included in the respective plot as thermostable virus controls. Table 1 lists the respective T r values and thermofluor assays were conducted in triplicate.

Prior to carrying out stability assays, viruses were treated with chloroform to remove adventitious agents containing a lipid envelop or membrane, and then amplified using the ZZ-R 127 goat epithelium cell line which expresses the principle FMDV receptor integrin αvß6 [8]. Viruses were then purified by sucrose gradient centrifugation [9], and used to perform thermofluor-based stability assays [4]. This method uses a dye to monitor the temperature (*T*_r_) at which the viral genome is released as a readout for capsid dissociation. To facilitate the comparison of capsid stability between all strains, thermofluor assays were performed in 1x PBS in the absence of divalent cations which effect the thermostability of the FMDV capsid in a serotype-specific manner [4]. Dissociation temperatures were successfully obtained for all viruses belonging to each of the four serotypes (Table 1 and Supplementary Fig. 2).

Within each serotype, individual strains exhibited different thermostabilities; T_r_ values ranged from 47.0 °C to 52.0 °C for the O serotype, from 48.0 °C to 52.3 °C for the A serotype, from 39.4 °C to 47.2 °C for the SAT1 serotype and from 45.3 °C to 50.1 °C for the SAT2 serotype. These results show the diversity in stability between and within serotypes, but also facilitated a key finding that has not been reported to date; that is, all the A serotype viruses analysed from East Africa were comparatively unstable. Until now it has been considered that the FMDV A serotype is the most stable. This is due to the higher levels of stability exhibited by strains such as A/IRAQ/22/1995 [10], [11]. Therefore, to confirm these results four other FMDV A strains, A/IRAQ/22/1995, A/IRAN/1/2005, A/ARG/1/2000 and A/SAU/1995 were prepared and analysed alongside the East African A strains; indeed, all four non-African A strains were comparatively more stable than the East African strains, exhibiting T_r_ values of 55.0 °C ± 0.0, 55.2 °C ± 0.3, 56.0 °C ± 0.0 and 55.7 °C ± 0.3, respectively (Table 1, Supplementary Fig. 2B).

### 3.3 Accelerated stability studies confirm thermostability of selected candidates

For each of the four FMDV serotypes, a single stable strain (O/ETH/29/08, A/ETH/9/08, SAT1/KEN/80/10, SAT2/ETH/65/09) was selected as a candidate seed stock and taken forward for stability confirmation assays, cell adaptation, long-term storage analysis and serology studies.

**Fig. 1:**
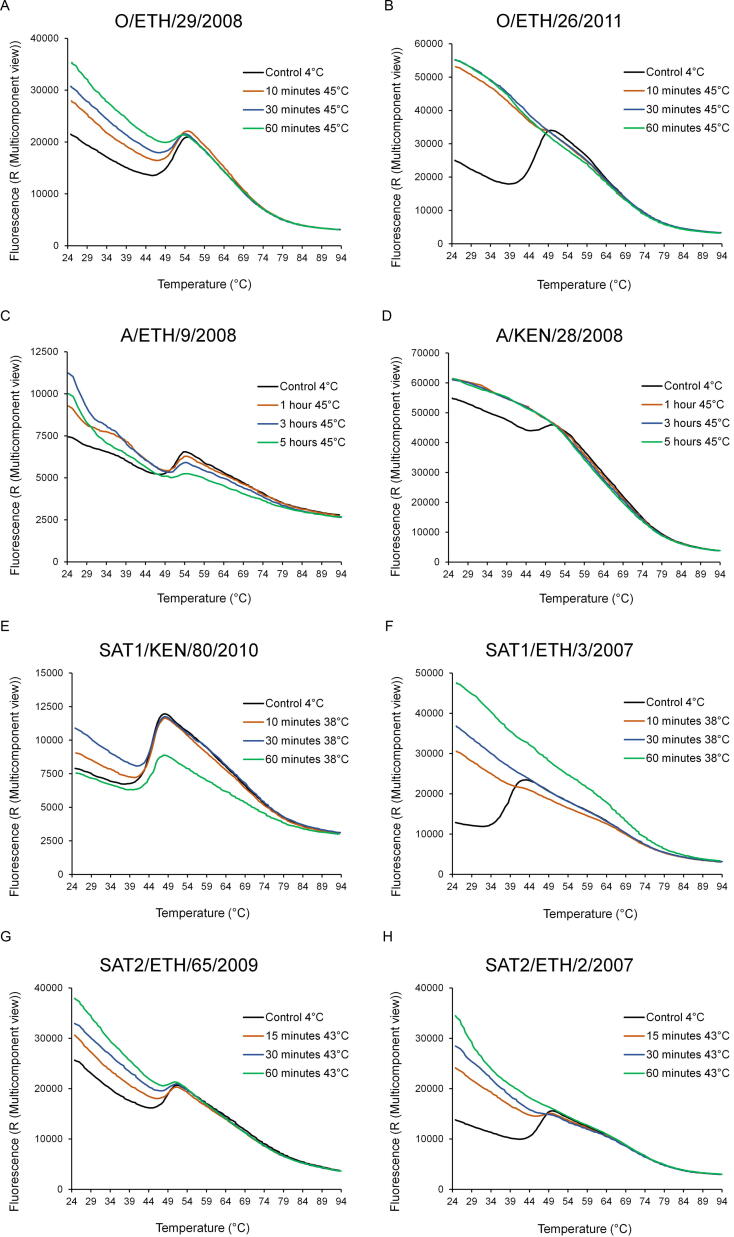
Accelerated thermostability assessment of candidate FMDV vaccine strains. Thermostability of the four candidate vaccine strains (O/ETH/29/2008, A/ETH/9/2008, SAT1/KEN/80/2010 and SAT2/ETH/65/2009) was confirmed by exposure to an elevated temperature prior to thermofluor analyses. An equal amount of control virus, with lower thermostability and belonging to the same serotype (O/ETH/26/2011, A/KEN/28/2008, SAT1/ETH/3/2007 and SAT2/2/2007, respectively), was analysed alongside each candidate strain. Virus strains belonging to the O and A serotypes were exposed to 45 °C, SAT1 to 38 °C and SAT2 to 43 °C for the indicated period before being tested by thermofluor assay. The loss of a dissociation peak, as observed for each control virus after 1 h heat treatment, indicates the absence of intact capsid due to its dissociation. Assays were conducted in triplicate.

To further validate the stability of the four selected FMDV strains we carried out accelerated stability assays alongside the least stable strain identified for each serotype. These experiments involved exposing virus to an elevated temperature for different periods of time prior to thermofluor analysis. In addition to the four selected stable strains, equal amounts of four non-stable strains (O/ETH/26/2011, A/KEN/28/2008, SAT1/ETH/3/2007 and SAT2/ETH/2/2007) identified in the thermostability screens were used as controls; strains belonging to the O and A serotypes were exposed to 45 °C, SAT1 to 38 °C and SAT2 to 43 °C. For each of the four serotypes, the respective reduction in thermofluor signal (reduction in dissociation peak) at each time point was then used to compare the level of capsid dissociation between the selected stable and less stable control strains (Fig. 1). In comparison to the respective stable virus, the unstable strain of each serotype exhibited a complete loss of dissociation peak following one hour of heat treatment (Fig. 1B, D, F, H). Although a reduction in the height of the dissociation peak for each of the stable strains was observed, the retention of a dissociation peak over the course of heat treatment indicated the presence of intact virus (Fig. 1A, C, E, G). Accelerated stability studies were performed over the course of one hour for strains belonging to the O, SAT1 and SAT2 serotypes. Likewise, the A serotype strains were also tested over a heat treatment period of one hour, but as no clear reduction in dissociation peak was observed for A/ETH/9/08, longer durations of heat treatment were then performed. Surprisingly, a dissociation peak indicative of intact virions was still observed after five hours of heat treatment at 45 °C (Fig. 1C).

We have previously reported the use of the thermofluor assay to improve vaccine formulation and shown that the presence of divalent cations and sucrose can have a dramatic effect on stability of the FMDV capsid [4]. To examine the effects of such excipients on the four candidate strains we next performed thermofluor assays using inactivated virus in the presence of divalent cations (calcium and magnesium) and with or without sucrose. Additional thermofluor assays were conducted to determine the stability of each virus in cell culture media, DMEM/F12 (containing 121 mM NaCl, 1 mM CaCl_2_) used to grow goat cells and GMEM (containing 110 mM NaCl and 1.8 mM CaCl_2_) used to grow BHK-21 cells. As expected, the conditions used for the thermofluor assays had differing effects on the thermal stability of the candidate strains (Table 2). Surprisingly, the addition of relatively low levels of calcium (20 µM) and magnesium (10 µM) were sufficient to increase the thermostability of each virus. In comparison to the other viruses, the inactivated SAT1/KEN/80/10 and O/ETH/29/08 strains exhibited the widest range in thermal stability (9 °C and 8.5 °C, respectively); their highest stability (*T*_r_ 53.0 °C ± 0.1 and 59.5 °C ± 0.0, respectively) was observed in DMEM/F12 media. Similarly, inactivated SAT2/ETH/65/09 exhibited its highest thermal stability (*T*_r_ 55.0 °C ± 0.2) in DMEM/F12 media, whilst inactivated A/ETH/9/08 exhibited its highest thermal stability (*T*_r_ 54.5 °C ± 0.2) in both DMEM/F12 media, as well as PBS containing calcium, magnesium and sucrose.

**Table 2:** Effect of excipients on thermostability of inactivated vaccine antigens. The thermostability of each inactivated vaccine antigen was assessed in PBS in the absence or presence of the indicated excipients (calcium (20 µM), magnesium (20 µM), sucrose (6% w/v)), or in DMEM/F12 or GMEM medium. The respective capsid dissociation temperature (*T*_r_) is shown for each condition. The *T*_r_ of live FMDV assayed in PBS is shown for comparison. Thermofluor assays were performed in triplicate.

### 3.4 Stability analyses following long term storage

**Fig. 2:**
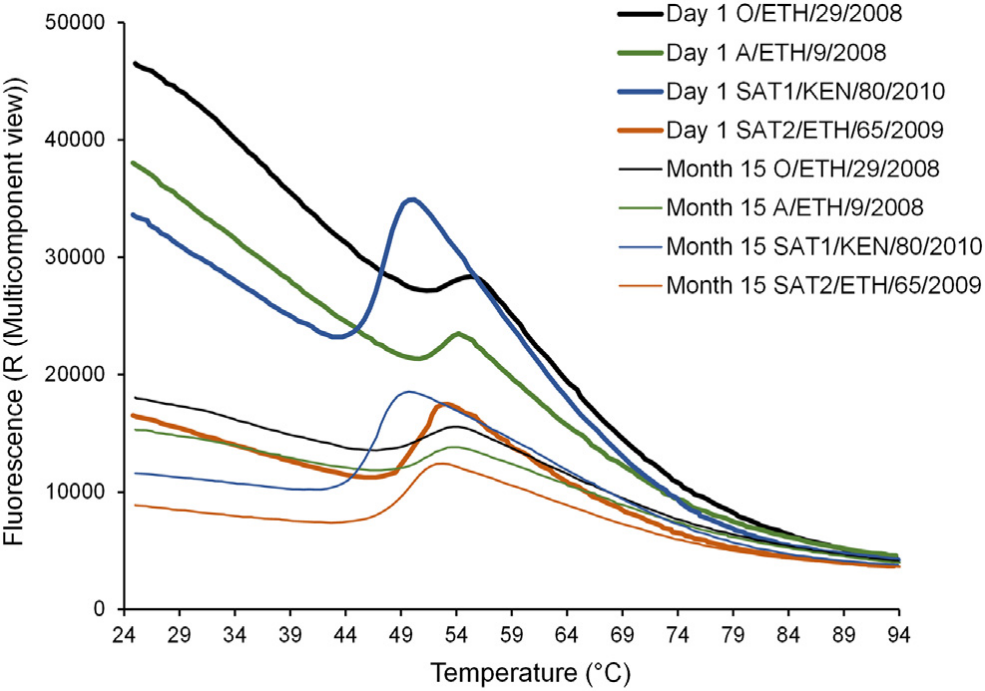
Storage assessment of inactivated vaccine antigens. Unformulated, inactivated candidate vaccine strains (O/ETH/29/2008, A/ETH/9/2008, SAT1/KEN/80/2010 and SAT2/ETH/65/2009) were stored at +4 °C for 15 months, during which the integrity of their capsid was analysed by thermofluor assay (see Table 3). Day 1 and month 15 thermofluor analyses are shown to facilitate comparison of the respective dissociation peaks at the beginning and end of the storage period, respectively. Thermofluor assays were performed in triplicate.

**Supplementary Fig. 3:**
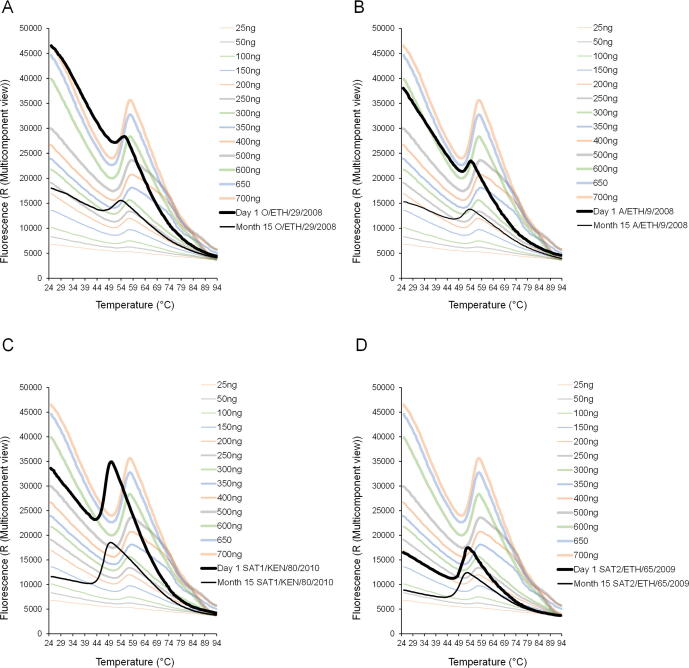
Estimation of intact antigen loss following long-term storage at 4 °C. Unformulated, inactivated candidate vaccine strains (O/ETH/29/2008, A/ETH/9/2008, SAT1/KEN/80/2010 and SAT2/ETH/65/2009) were stored at +4 °C for 15 months, during which the integrity of their capsid was assessed by thermofluor assay (see Table 3 and Figure 2). To estimate the loss of intact SAT1 antigen over the storage period, the respective dissociation peaks generated using day 1 and month 15 stored antigen were compared with peaks generated using different amounts of virus under the same assay conditions. Negligible losses for stored intact O, A and SAT2 antigens, and a 20% reduction of intact SAT1 antigen was estimated. Thermofluor assays were performed in triplicate.

To investigate the effects of long-term storage on stability, the four inactivated antigen were stored for a period of 15 months at 4 °C in PBS buffer containing Ca and Mg cations. Thermofluor analyses of inactivated antigen samples during the period of storage identified small decreases in their respective *T*_r_ values (0.7–1.7 °C), but revealed dissociation peaks, confirming the presence of intact antigen (Table 3 and Fig. 2). Each of the O, A and SAT2 inactivated antigens showed comparable dissociation peaks at the beginning (day 1) and the end (month 15) of storage, revealing negligible levels of dissociation had occurred. Similar comparison of SAT1 inactivated antigen revealed a reduction in dissociation peak at the end of storage, suggesting some dissociation of capsid had occurred. In our hands, a dissociation peak can be detected using 50 ng of intact antigen to perform the thermofluor assay, above which a positive correlation is observed between amount of intact capsid and height of dissociation peak. To estimate the loss of intact SAT1 inactivated antigen over the storage period, we compared the dissociation peaks generated using day 1 and month 15 stored antigen with peaks generated using different amounts of antigen under the same conditions (Supplementary Fig. 3). Using this method, we estimated the reduction of intact SAT1 to be approximately 20%.

**Table 3:** Storage assessment of inactivated vaccine antigens. Unformulated, inactivated vaccine antigens were stored at +4 °C for 15 months, during which the integrity of their capsid was analysed by thermofluor assay at the indicated time points. The respective capsid dissociation temperature (*T*_r_) is shown for each time point. Thermofluor assays were performed in triplicate. Fig. 2 shows dissociation plots for day 1 and month 15 analyses.

### 3.5 Cell culture adaptation of selected FMD vaccine strains

Large scale production of FMD vaccines uses suspension BHK-21 cells to amplify FMDV prior to inactivation and downstream processing of the antigen, necessitating candidate vaccine strains to be cell-culture adapted. To cell culture adapt the four candidate strains, we serially passaged each virus using BHK-21 cells until cytopathic effect was observed; A/ETH/9/08 and SAT1/KEN/80/10 were passaged six, SAT2/ETH/65/09 seven and O/ETH/29/08 eight times. Following cell culture adaptation, titres of ≥1 × 10^7^ plaques/ml were routinely obtained when virus was propagated (MOI of 0.05) for small scale vaccine production in confluent monolayers of BHK-21 for 24 h (data not shown). To identify changes resulting from cell culture adaptation of each strain, we sequenced the region of viral genome that encodes the outer structural proteins VP1-3 of both non-cell culture adapted as well as cell culture adapted O, A, SAT1 and SAT2 FMDV. Two changes, VP2_132_ M to I and VP3_205_ V to A, were observed for O/ETH/29/08, one change, VP2_82_ E to K, for A/ETH/9/08, and three changes, VP1_111_ N to K, VP1_205_ D to N and VP3_9_ D to A for SAT1/KEN/80/10. No changes were observed in VP1-3 for SAT2/ETH/65/09 following cell culture adaptation.

### 3.6 Validation of immunogenicity and strain suitability for use as vaccines in East Africa

**Fig. 3:**
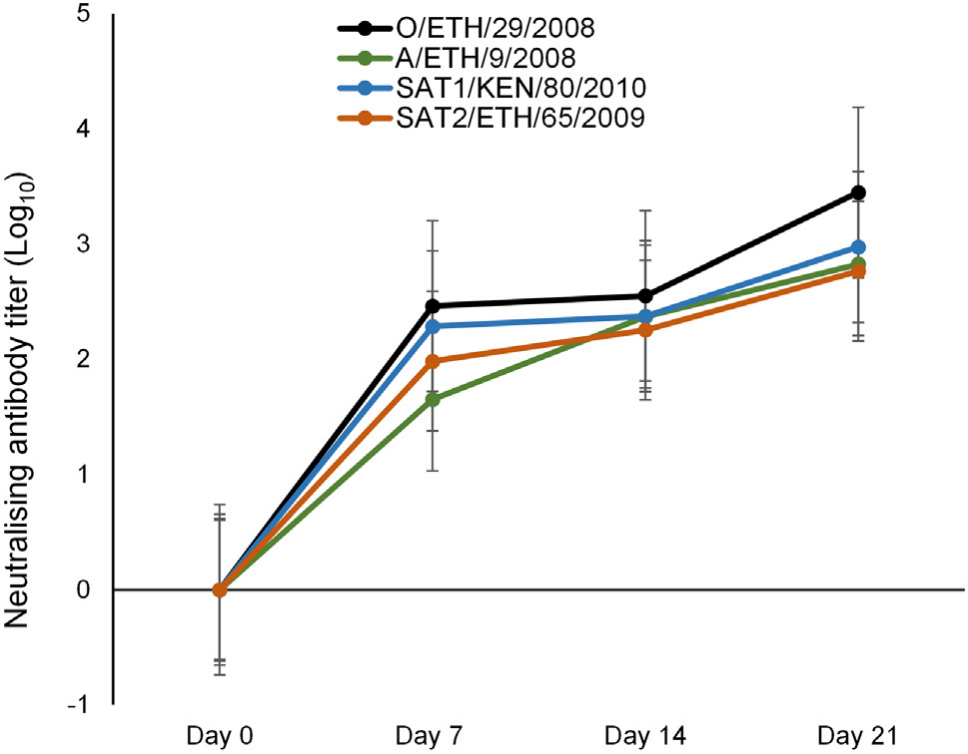
Production of monovalent vaccinate sera. Separate groups of five calves were vaccinated with one of the candidate FMD vaccines (O/ETH/29/2008, A/ETH/9/2008, SAT1/KEN/80/2010 and SAT2/ETH/65/2009) on day 0 and boosted on day 14 post vaccination; blood samples were collected 0, 7-, 14-, and 21-days post vaccination and assayed to determine homologous mean virus neutralizing-antibody titres (VNT (log10)). Error bars indicate standard deviation.

To assess the ability of each of the candidate strains to elicit the production of neutralising antibodies, groups of five calves were individually vaccinated with equal quantities of intact inactivated antigens. A second vaccination, using the same vaccine preparations that had been stored at 4 °C, were administered on day 14 of the study. Serum samples from days 0, 7, 14 and 21 post vaccination were subsequently used to determine virus-neutralizing-antibody titres (VNT) against the homologous FMDV strain. Homologous VNT confirmed that neutralizing antibody titres >2 log_10_ (considered protective) had been elicited by each vaccine on days 14 (pre-boost) and day 21 (post-boost) (Fig. 3) [12]. To evaluate the candidate vaccine strains in the context of their suitability to elicit a protective response against other available East African strains belonging to the same serotype, heterologous VNTs were conducted using the panel of FMDV strains (listed in Table 1) screened for thermostability (Table 4). Mean r_1_ values >0.3, the cut off value used in vaccine matching to predict antigenic similarity between a vaccine strain and a target strain, were observed for 10 of the 12 tested FMDV O strains, with high reciprocal neutralisation values (≥32) being observed in all tests [13], [14] (Table 4). Mean r_1_ values >0.3 were observed for 6 of the 7 A FMDV strains that were tested; however, it should be noted that sera from Animal 1–3 exhibited low reciprocal neutralisation titres and although mean r_1_ values >0.3 were observed for A/KEN/28/2008 and A/SUD/7/2011, only two out of the five sera exhibited reciprocal neutralisation values ≥32 (Table 4). With regards to the SAT serotypes, 5 of the 6 SAT1 and 7 of the 8 SAT2 FMDV strains exhibited a mean r_1_ value >0.3. Of note, three of the five test sera yielded reciprocal neutralisation values of 32 for SAT1/ETH/21/2007. A mean r_1_ value >0.3 was also observed for the A/SAU/1995 control virus.

**Table 4:** Serological analyses using vaccinate sera raised against inactivated vaccine antigens. Day 21 monovalent vaccinate sera raised against the four candidate vaccine strains (O/ETH/29/2008, A/ETH/9/2008, SAT1/KEN/80/2010 and SAT2/ETH/65/2009) were used to determine heterologous virus neutralizing-antibody titres for the FMDV strains listed in Table 1. For each challenge virus, the reciprocal neutralizing-antibody titre of individual serum from 5 vaccinates is shown, along with the mean r_1_ value determined using the respective mean (mean r_1_ = mean heterologous reciprocal neutralizing-antibody titre/mean homologous neutralizing-antibody titre). Mean r_1_ values have been heat mapped as indicated. The mean homologous titre (bold and yellow highlighted) first-listed above each challenge virus was used to determine the respective r_1_ value.

**Supplementary Fig. 4:**
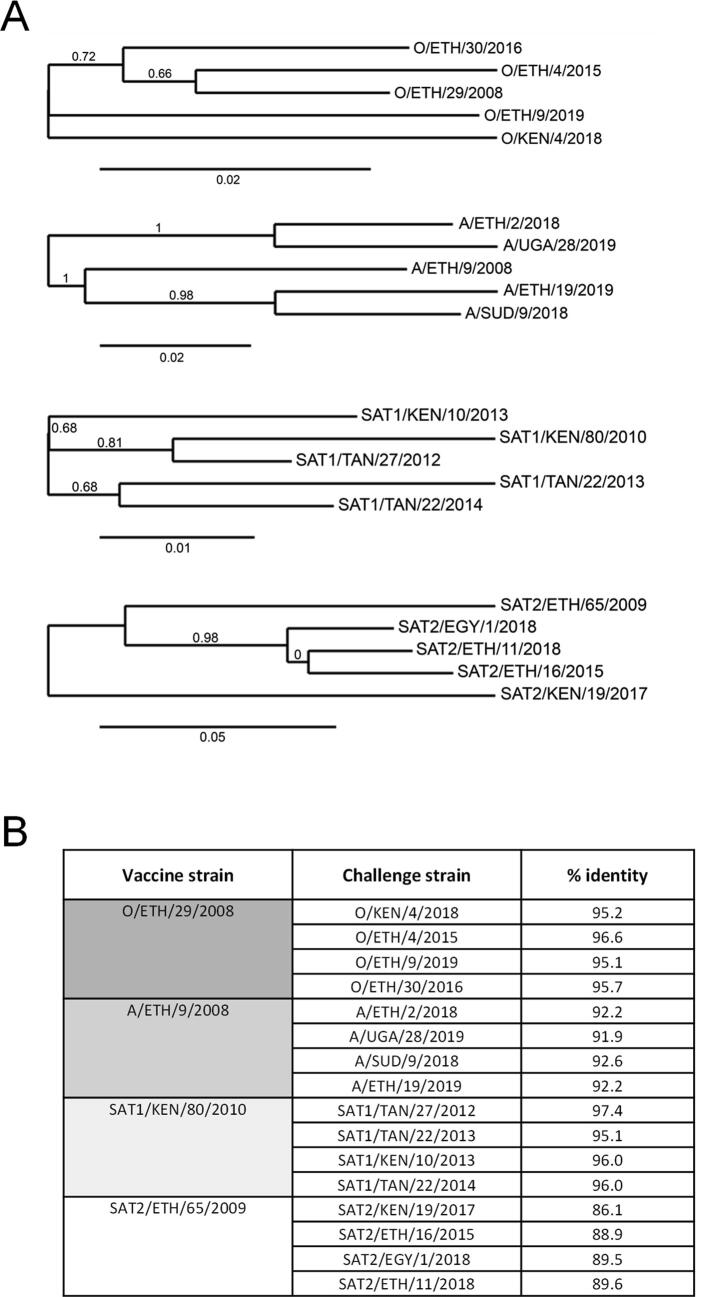
Comparison of the external capsid amino acid sequences of candidate vaccine strains with Eastern Africa FMDV reference antigen panel. (A). On-line software (NGphylogeny.fr) was used to analyse the amino acid sequences of the VP1-3 external capsid proteins from each of the four candidate vaccine strains (O/ETH/29/2008, A/ETH/9/2008, SAT1/KEN/80/2010 and SAT2/ETH/65/2009) alongside the respective Eastern African FMDV reference strains (4 for each of the O, A, SAT1 and SAT2 serotypes) selected by the World Reference Laboratory for Foot-and-Mouth Disease (WRL-FMD). (B) Percent identity of VP1-3 amino acids from each of the four candidate vaccine strains and the respective reference strain determined using on-line software (MUSCLE).

Immunogenicity of the vaccine candidates was also validated against the Eastern Africa FMDV Reference Antigen Panel established by the WRL-FMD (Table 5). For the O, SAT1 and SAT2 candidate vaccine strains, day 21 sera from all five animals conferred protective titres (VNTs >32) against each of the respective FMDV isolates in the Reference Antigen Panel. Similarly, for the A candidate vaccine strain, day 21 sera from four of five animals conferred neutralizing titres >32 against each of the four challenge viruses; sera from the fifth animal exhibited neutralizing titres >32 for three of the four respective challenge viruses. These results show the identified candidate strains stimulate humoral responses that are predicted to protect against FMDV recently circulating in East Africa. Comparison of the amino acid sequences of the external capsid proteins VP1-3 showed that the candidate vaccine strains are closely related to the antigen panel and share high levels of identity across the external VP1-3 capsid proteins (Supplementary Figure 4).

**Table 5:** Serological analyses to assess strain suitability for vaccine use in East Africa. Day 14 and 21 monovalent vaccinate sera raised against the four candidate vaccine strains (O/ETH/29/2008, A/ETH/9/2008, SAT1/KEN/80/2010 and SAT2/ETH/65/2009) were used to determine heterologous virus reciprocal neutralizing-antibody titres for the respective reference strains (4 indicated strains for each of the O, A, SAT1 and SAT2 serotypes) selected by the World Reference Laboratory for Foot-and-Mouth Disease (WRL-FMD; Eastern Africa FMDV Reference Antigen Panel). Reciprocal neutralising-antibody titres have been heat mapped as indicated.

## 4 Discussion

To successfully carry out immunization programs, ensuring stability of vaccines is crucial. In this context, two issues are critical for successful FMDV vaccine campaigns, namely: 1) predicting and assessing vaccine stability and 2) establishing and maintaining an appropriate cold chain. The capsids of inactivated preparations of the O and SAT serotypes are particularly susceptible to dissociation at elevated temperature [4], [10] and the implementation of a cold chain in many parts of East Africa, which experience a hot climate year-round, is often problematic. To address these issues, we have used a novel approach to identify naturally stable candidate FMDV vaccine strains for use in East Africa; this involved assessing the thermostability of recently circulated O, A, SAT1 and SAT2 FMDV strains. Specifically, we used the thermofluor assay as a screening tool to rapidly determine the temperature at which the FMDV capsid dissociates under defined assay conditions; this method has been developed as a system to examine FMDV capsid thermostability and we have shown a positive correlation between dissociation temperature and capsid stability at lower temperatures [4], [11], [15], [16]. Accelerated thermostability assays were used in this study to confirm the selected strains confer an advantage at elevated temperatures in comparison to less stable strains that were identified. Interestingly, a myriad of capsid stabilities was identified, not only between O, A, SAT1 and SAT2 serotypes, but also between strains within these serotypes. For each of the four serotypes investigated, capsid dissociation temperatures ranged by at least 4 °C amongst the strains that were tested, whilst SAT1 exhibited a 7.8 °C range. Although SAT1/KEN/80/2010 was selected, SAT1/TAN/12/2012 exhibited a higher T_r_ (45.5 ± 0.0 vs 47.2 ± 0.3, respectively) and is therefore a potential vaccine candidate; indeed, the WRL-FMD SAT1 reference panel includes three SAT1 strains (Supplementary Figure 4) belonging to the same lineage. Surprisingly, we show herein that East African FMDV A strains exhibit a variety of thermostabilities, and that in comparison to available non-East African strains they are less stable. To date, the A serotype has been perceived as the most stable in comparison to the other serotypes, and the underlying reasons for the disparity in thermostability between non East-African and East-African A strains are not known but may involve selective genome pressures such as target host species and environmental factors.

Our previous research focused on the use of reverse genetics to artificially increase stability of pentamer interfaces in the FMDV capsid, and although this approach has merits, manipulation of the FMDV capsid has the potential to affect both antigenicity and capacity to replicate in cell culture [11], [16]. Future work will focus on investigating targeted capsid mutations that confer an increase in stability in the genetic background of naturally stable strains [11]. We have already shown that the stability of the SAT2 (SAT2/ETH/65/09) candidate vaccine strain identified in this study can be further improved by the insertion of the external VP1-3 capsid proteins of SAT2 into the genetic background of an FMDV O strain [15]. The basis for this involves the internal VP4 capsid protein, presumably through its interaction with the packaged viral genome. Importantly, the recombinant SAT2 virus showed growth kinetics equal to that of the wild type SAT2 virus with better thermostability.

In addition to the stability of candidate seedstocks, there are a number of challenges that impede the development of efficacious vaccines for East Africa. Cell culture adaptation can be time consuming and require pro-longed periods of passaging to overcome sub-optimal virus yields; in turn this can alter antigenicity and prevent the rapid establishment of new master seedstocks. Such cell culture adaptation often involves the accumulation of positive charges on the capsid to facilitate interaction with the heparan sulfate receptor [17]. The strains selected in this study were able to be cell culture adapted to BHK-21 cells following a low number of passages and high virus yields were obtained (1 × 10^7^ plaques/ml) during small scale vaccine production. Sequence analyses of the external capsid proteins (VP1-3) for each strain identified a small number of amino acid changes in the O, A and SAT1 vaccine candidates and none in the SAT2 candidate. Four of the six amino acid changes in VP1-3 that were identified in this study following cell culture adaptation have been reported for the respective serotype [17]; a VP2_132_ V to I cell culture adaptation (instead of VP2_132_M to I described herein) has been reported for serotype O [18], a VP2_82_ E to K adaptation for serotype A [19], and VP1_111_ N to K and VP3_9_ D to A adaptations for serotype SAT1 [20], [21]. Importantly, the changes accrued during cell culture adaptation did not affect capsid stability (data not shown).

An essential vaccine property is the ability to elicit a protective immune response against both the homologous FMDV strain and heterologous strains that have recently circulated. Predicting heterologous cross-protection using antibody titres can be problematic due to the limited number of heterologous studies that have been performed, strain differences in the correlation between titre and protection, and inter-laboratory assay variation. To assess cross-protection elicited by the candidate strains, we initially used the respective vaccinate sera to perform VNT against the panel of strains listed in Table 1. These analyses were subsequently extended following submission of the sera to the WRL-FMD for independent VNT against a panel of strains tailored to cover the genetic diversity within the FMDV lineages that circulate in Eastern African countries and selected to assess the suitability of vaccines for use in Eastern African countries. The panel is comprised of viruses from Burundi, Democratic Republic of Congo, Eritrea, Ethiopia, Kenya, Rwanda, Somalia, South Sudan, Tanzania, and Uganda and includes the following lineages: O/EA-2, O/EA-3, O/EA-4, A/AFRICA/G-I, A/AFRICA/G-IV, SAT1/I, SAT2/IV, and SAT2/VII. Recent recommendations by WRL-FMD to AgResults (Cross-neutralisation measure AgResults Final v2.1.pdf (wrlfmd.org)) on the use of serological indicators of cross-protection suggests the use of sera collected 21 days after one vaccination to perform VNT and a log_10_ reciprocal titre at or above 1.5 (reciprocal titre of ≥32) for three out of five cattle as an indicator of heterologous cross-protection. Although the day 21 sera raised in this study were collected after two vaccinations, these VNT showed neutralizing titres ≥32 for all samples. Combined with their increased stability and adaptation to BHK-21, these serology results support continuation of the development of the candidate strains for use in East Africa. Indeed, the vaccines used herein are not the final products, and additional dose-titration work may achieve improved neutralisation titres, particularly for the A serotype which exhibited variability between vaccinates.

Future work will investigate the application of the four candidate strains as a quadrivalent vaccine product, with focus on the analyses of sera collected at 21 days after a single vaccination. Taken together, this work highlights the importance of tools to predict and assess FMDV vaccine stability, and in combination with cell culture adaptation and serological tests describes a feasible approach to rapidly screen and select the most appropriate vaccine strain.

## CRediT authorship contribution statement

**Ben Jackson:** Validation, Formal analysis, Investigation, Data curation, Writing - review & editing. **Yongjie Harvey:** Validation, Formal analysis, Investigation, Data curation. **Eva Perez-Martin:** Formal analysis, Investigation. **Ginette Wilsden:** Formal analysis, Investigation. **Nicholas Juleff:** Conceptualization, Writing - review & editing. **Bryan Charleston:** Conceptualization, Writing - review & editing, Funding acquisition. **Julian Seago:** Conceptualization, Methodology, Validation, Formal analysis, Investigation, Resources, Data curation, Writing - original draft, Writing - review & editing, Visualization, Supervision, Project administration, Funding acquisition.

## Declaration of Competing Interest

The authors declare that they have no known competing financial interests or personal relationships that could have appeared to influence the work reported in this paper.
